# Antioxidant, Antidiabetic, and Antibacterial Potentials and Chemical Composition of *Salvia officinalis* and *Mentha suaveolens* Grown Wild in Morocco

**DOI:** 10.1155/2022/2844880

**Published:** 2022-06-15

**Authors:** Samiah Hamad Al-Mijalli, Hamza Assaggaf, Ahmed Qasem, Adel G. El-Shemi, Emad M. Abdallah, Hanae Naceiri Mrabti, Abdelhakim Bouyahya

**Affiliations:** ^1^Department of Biology, College of Sciences, Princess Nourah Bint Abdulrahman University, P.O. Box 84428, Riyadh 11671, Saudi Arabia; ^2^Laboratory Medicine Department, Faculty of Applied Medical Sciences, Umm Al-Qura University, Makkah 21955, Saudi Arabia; ^3^Department of Science Laboratories, College of Science and Arts, Qassim University, Ar Rass 51921, Saudi Arabia; ^4^Laboratory of Pharmacology and Toxicology, Bio Pharmaceutical and Toxicological Analysis Research Team, Faculty of Medicine and Pharmacy, University Mohammed V in Rabat, Rabat, BP 6203, Morocco; ^5^Laboratory of Human Pathologies Biology, Department of Biology, Faculty of Sciences, Mohammed V University in Rabat, Rabat, Morocco

## Abstract

This work evaluated in vitro antioxidant, antidiabetic, and antibacterial properties of *Salvia officinalis* (*S*. *officinalis*) and *Mentha suaveolens* (*M*. *suaveolens*) essential oils (EO). The EOs were extracted, and their chemical composition was determined using GC-MS analysis. The in vitro antioxidant, antidiabetic, and antibacterial activities of *S*. *officinalis* and *M*. *suaveolens* EO were shown to be remarkable. Furthermore, *S*. *officinalis* EO demonstrated better antioxidant findings (using DPPH, ABTS, and FRAP test) than *M*. *suaveolens* EO (*p* < 0.5). There were no significant differences in the inhibitory effects of the EOs on *α*-amylase and *α*-glucosidase activities in the antidiabetic assays. All of the examined bacterial strains (10 different strains), with the exception of *P*. *aeruginosa*, demonstrated significant sensitivity to the tested EOs, with *M*. *suaveolens* EO exhibiting better activity than *S*. *officinalis* EO. Thus, the research indicated that EO from these two medicinal plants has considerable potential for application in the formulation of antibacterial, antioxidant, and antidiabetic pharmaceuticals. However, more research studies are required to interpret the pharmacologic action of the studied EOs and their principal constituents and to confirm their safety.

## 1. Introduction

About 80% of rural people in developing countries use traditional medicine made from plants. Even people in developed countries are becoming more interested in medicinal plants [1]. Indeed, over 25% of medications used in the previous two decades are typically extracted from plants, while the remaining 25% are chemically altered natural substances. Despite this, only around 5%–15% of the roughly 250,000 higher plants have ever been studied for pharmacological activities [2]. Numerous studies conducted over the last few decades have shown that research on therapeutic plants is critical and bioactive phytochemicals or bionutrients are abundant in medicinal plants; these phytochemicals have a critical role in avoiding chronic illnesses such as cancer [3–5], diabetes [6–9], and coronary heart disease [10–12]. Dietary fiber, detoxifying agents, antioxidants, anticancer, neuropharmacological agents, and immune-stimulating agents are the key groups of phytochemicals having disease-preventive properties [13].

There is now strong evidence that oxidative stress caused by free radicals or reactive oxygen species is the main cause of a number of human neurological diseases. Antioxidant compounds found in foods and medicinal plants can be used as chemo-preventive agents [14]. Also, oxidative stress is a significant factor for development of diabetes mellitus. Natural antioxidants are abundant in plant-based foods. Numerous epidemiological and clinical research, as well as in vivo and in vitro studies, have shown that fruits with varying phytochemical profiles have the capacity to normalize blood glucose levels [15]. Diabetes mellitus is becoming a severe hazard to human health in all regions of the globe due to its fast-rising prevalence. Additionally, during the last several years, some novel bioactive compounds derived from plants have shown superior anti-diabetic action than commonly used oral hypoglycemic medications [16].

Antibiotics' capacity to treat microbial infections has deteriorated over the last several decades, and multidrug resistant bacteria have arisen; as a result, medicinal plants are recommended as a source of novel antibacterial compounds such as coumarin molecules which were reported as promising antibacterial agents [17]. It is believed that taking corrective and preventive action now through collaborative and innovative approaches in the field of novel antibacterial drug discovery and development is critical for preparing for future pandemics caused by multidrug-resistant bacteria, a target that truly deserves our full attention [18]. Interestingly, numerous studies revealed that there are hundreds of medicinal plants that documented antibacterial effectiveness which may be turned into effective medications [19]. According to scientific reports, the antibacterial activity of some medicinal plants is a consequence of their phytochemical constituents, which include sulfur-containing compounds, alkaloids, terpenoids, carotenoids, and polyphenols [20].

Sage (*Salvia officinalis* L.) is an aromatic and medicinal plant that belongs to the Lamiaceae family. This family has about 900 species that are found around the globe, with some of them having a high economic value due to their usage in the cosmetic industry and perfumery [21]. *Salvia* is extensively used in traditional medicine as an antiseptic, antiscabies, antibacterial, antisyphilis, and anti-inflammatory medication, and it was reportedly used to cure fever and some digestive disorders in several locations of the Middle East [22].

Apple mint (*Mentha suaveolens* Ehrh.) is a perennial rhizomatous plant, the genus *Mentha* is a member of the Labiatae family and is well-known for its high essential oil concentration, and this herb is widespread in a number of Mediterranean countries [23]. Labiatae family has over 260 genera and more than 7000 species, and it has been widely known since ancient civilizations [24]. It has been used in traditional medicine in Mediterranean countries for a wide variety of purposes, including cardiovascular effects, antibacterial properties, analgesic properties, anti-inflammatory properties, and antiviral properties [25].

Although various studies on the bioactive properties of *S*. *officinalis* and *M*. *suaveolens* have been conducted worldwide, the essential oils (EOs) of these plants that grow wild in Morocco have received little attention in terms of their biological effects. Moreover, the current investigation aimed to determine the chemical composition of *S*. *officinalis* EOs (SOEO) and *M*. *suaveolens* EOs (MSEO) and to evaluate their antioxidant, antidiabetic, and antibacterial potentials.

## 2. Materials and Methods

### 2.1. Chemicals and Reagents

Acarbose, ascorbic acid, 2, 2′-diphenyl-1-picrylhydrazyl (DPPH), 6-hydroxy2, 5, 7, 8-tetramethylchroman-2-carboxylic acid (Trolox), 2, 2-azino-bis-3-ethylbenzothiazoline-6-sulfonic acid (ABTS), *p*-nitrophenyl-*α*-D–D-glucopyranoside (p-NPG), and 3, 4-dihydroxy phenylalanine (L-DOPA) were procured from Sigma-Aldrich (France). *α*-Amylase (from *Bacillus licheniformis*) and *α*-glucosidase (from *Saccharomyces cerevisiae*) were purchased from Roche Diagnostics (USA). All other reagents used were of analytical grade.

### 2.2. Plant Materials and EOs Extraction

During April 2021, the aerial parts of *S*. *officinalis* and *M*. *suaveolens* were manually collected from their natural habitat in the Taza region of Morocco. The plants were identified according to the procedure described by González-Tejero et al. [26] and confirmed by the botanists at the Botany Department of the Scientific Institute of Rabat, University of Mohammed V Rabat, Morocco. Voucher specimens of each plant were deposited in the herbarium under the voucher specimen code RAB61862 for *S*. *officinalis* and RAB611848 for *M*. *suaveolens*. The extraction process of the EOs was carried out by hydrodistillation in a Clevenger-type apparatus. Briefly, 100 g of the dry powder of each plant were placed in a flask filled to 2/3 with water; the whole is brought to the boil for 3 hours. The oil is recovered and then stored at a temperature of 4°C.

### 2.3. Chemical Composition Analysis

The chemical components of SOEO and MSEO were determined by using gas chromatography/mass spectrometry (GC/MS) analysis conditions as described in our latterly published study [27].

### 2.4. In Vitro Antioxidant Assays

The antioxidant activity of the two tested EOs (solubilized in Tween 20 (5%)) was evaluated using the following three commonly used in vitro complementary assays: DPPH and ABTS radical scavenging assays, and ferric reducing/antioxidant power (FRAP) assay, and following the same procedures as described previously by our research group [28, 29] and by others. Each assay was carried out in triplicate, and Trolox and ascorbic acid were used as positive controls. In each assay, the concentrations of the EOs that provided 50% inhibition (IC_50_) were determined, and their values were presented in *μ*g/mL.

### 2.5. In Vitro Assessment of Antidiabetic Activity

The in vitro antidiabetic effects of SOEO and MSEO ((solubilized in Tween 20 (5%)) were determined by measuring their capacity to inhibit *α*-amylase and *α*-glucosidase enzymatic activity, following the same methods as we previously described [30], and the IC_50_ (*μ*g/mL) values were determined.

### 2.6. In Vitro Evaluation of Antibacterial Activity

#### 2.6.1. Bacterial Strains

In the present study, the antibacterial activity of SOEO and MSEO was carried out on ten referenced pathogenic bacterial strains divided as five Gram-positive strains including *Staphylococcus aureus*, *Staphylococcus epidermidis*, *Enterococcus faecalis*, *Listeria monocytogenes*, and *Bacillus cereus*, and five Gram-negative strains including *Escherichia coli*, *Salmonella typhimurium*, *Klebsiella pneumoniae*, *Proteus mirabilis*, and *Pseudomonas aeruginosa*. All the bacterial strains were brought from the Microbiology Lab at Mohammed V University in Rabat, after their identification using the standard microbiological procedures [31].

#### 2.6.2. Disc-Diffusion Test

The disc-diffusion test was employed to determine the preliminary antibacterial potential of SOEO and MSEO against the selected 10 bacterial pathogens, according to the protocol described previously by our research group [27]. Briefly, the tested EOs were mixed with 10% dimethyl sulfoxide (DMSO) to enhance their diffusion into the agar. Subsequently, a 0.5 McFarland (10^8^ CFU/mL) suspension of each studied bacterium was prepared in normal saline (0.9% NaCl) and inoculated through swabbing on Mueller–Hinton agar plates (Biokar, Beauvais, France). Then, 10 *μ*L of each EO was placed onto sterile paper discs with a diameter of 6 millimeters. Another disc holding 10 *μ*L of 10% DMSO was used as a negative control, and another disc containing chloramphenicol (30 g/disc) was employed as a referenced drug (positive control). Following that, all plates were incubated at 37°C for 24 hours, and the inhibition diameter was measured in millimeters (disk included) and reported as the mean ± standard deviation of three replicates.

#### 2.6.3. Determination of Minimum Inhibitory Concentration (MIC)

The broth dilution technique was used to determine the MIC values for SOEO and MSEO using a 96-well microtitration plate [32, 33]. Decreasing quantities of EOs were generated in microplates (final concentrations ranged between 20 and 0.039 mg/mL) in 10% DMSO. After adding 20 *μ*L of bacterial suspensions adjusted to 0.5 McFarland standard and 140 *μ*L of Mueller–Hinton broth, the microplates were incubated at 37°C for 24 hours. After incubation, 40 *μ*L of 2, 3, 5-diphenyltetrazolium chloride (TTC) (Sigma-Aldrich, Buchs, Switzerland) at a concentration of 0.2 g/mL was added and incubated for 30 minutes at 37°C. The existence of living bacteria is indicated by the presence of a red hue on TTC.

#### 2.6.4. Determination of Minimum Bactericidal Concentration (MBC)

The minimum bactericidal concentration (MBC) was determined for the two EOs against the examined bacteria as follows: 100 mL from each of the MIC tubes that exhibited no growth was subcultured on Mueller–Hinton agar plates and incubated at 37°C for 24 hours. MBC was defined as the lowest concentration that demonstrated no single colony of bacteria. Moreover, MBC/MIC values were calculated to classify the antibacterial agent as bacteriostatic or bactericidal [33].

### 2.7. Statistical Analysis

All tests were performed in triplicate and the obtained results are expressed as mean ± SD. Data were analyzed using SPSS software version 21, and comparisons of the means were determined by one-way analysis of variance (one-way ANOVA) followed by the Tukey test. Values with *p* < 0.05 were considered statically significant.

## 3. Results

### 3.1. In Vitro Antioxidant Activity of SOEO and MSEO

The two EOs were investigated for their antioxidant capacity using three complementary tests: DPPH, ABTS radical scavenging capacity, and FRAP. As given in Table 1, both SOEO and MSEO had potent antioxidant activity, and SOEO showed the highest antioxidant ability as compared with MSEO (IC_50_ = 53.19 ± 1.12 *μ*g/mL, 69.48 ± 2.05 *μ*g/mL and 75.19 ± 1.80 *μ*g/mL vs. 93.67 ± 2.17 *μ*g/mL, 112.41 ± 3.18 *μ*g/mL, and 129.74 ± 2.11 *μ*g/mL for DPPH, FRAP, and ABTS assay, respectively; *p* < 0.5).

### 3.2. In Vitro Antidiabetic Activities of SOEO and MSEO

The antidiabetic activities of SOEO and MSEO were determined using the inhibitory effects on *α*-amylase and *α*-glucosidase enzymatic activities. The results are expressed as IC_50_ and given in Table 2. The two EOs inhibited the activities of the two tested enzymes at low concentrations compared with the used standard drug (acarbose). However, no significant differences were detected between the inhibitory effects of the two EOs (IC_50_ of *S*. *officinalis* EO = 81.91 ± 0.03 *μ*g/mL and 113.17 ± 0.02 *μ*g/mL vs. IC_50_ of *M*. *suaveolens* EO = 94.30 ± 0.06 *μ*g/mL and 141.16 ± 0.21 *μ*g/mL, for inhibiting *α*-amylase and *α*-glucosidase enzymatic activity, respectively) (Table 2).

### 3.3. In Vitro Antibacterial Activity of SOEO and MSEO

As given in Table 3, the 10 examined bacterial strains were significantly (*p* < 0.05) susceptible to the antibacterial activity of both SOEO and MSEO, with the exception of *Pseudomonas aeruginosa* which demonstrated weak susceptibility toward the two EOs and complete resistance to the reference antibiotic (chloramphenicol at 30 *μ*g/disc). It was noted also that MSEO had showed comparably greater antibacterial activity than SOEO (Table 3). Moreover, the examined Gram-positive bacteria were more susceptible to the antibacterial activity of the two EOs than the tested Gram-negative bacteria. Next, the antibacterial efficacies of the two EOs were determined by using MIC and MBC assays. As given in Table 4, the overall MIC and MBC values for MSEO against all the tested bacteria were significantly lower than those for SOEO, reflecting its higher bactericidal activity than the latter one. Finally, the MBC/MIC ratios for both EOs were between 1 and 2, and *Pseudomonas aeruginosa* showed sensitivity to chloramphenicol only at its high concentration of 64 *μ*g/mL (Table 4), indicating the high antimicrobial-resistance property of this pathogenic bacterial strain.

### 3.4. Chemical Composition of SOEO and MSEO

**Figure 1:**
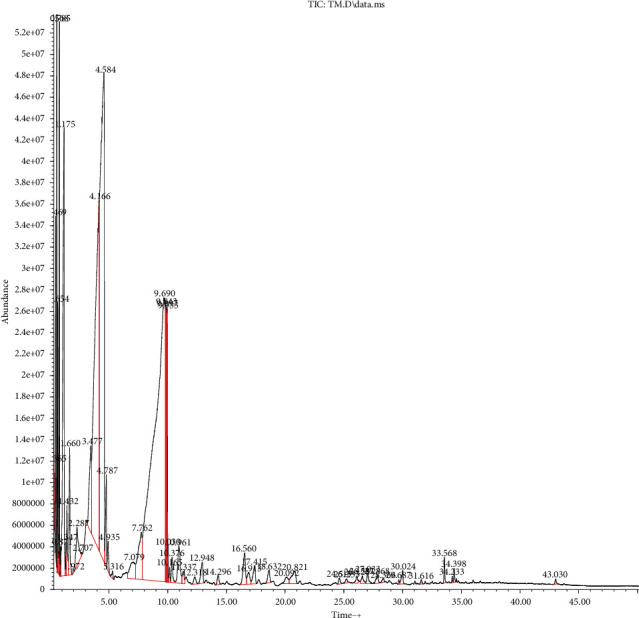
Gas chromatography of *S*. *officinalis* essential oils.

**Figure 2:**
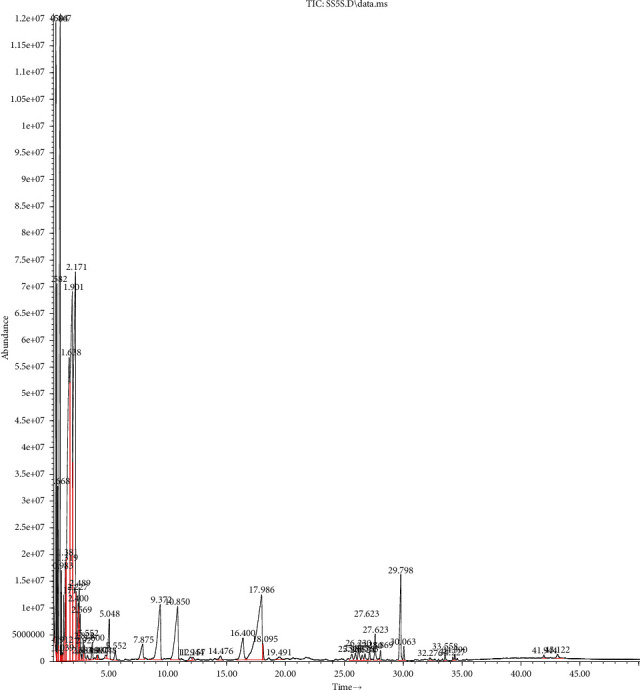
Gas chromatography of *Mentha suaveolens* .

As given in Table 5 and Figures 1 and 2, the principal compounds detected in SOEO were thujone (33.77%), caryophyllene (12.28%), humulene (12.19%), camphor (11.52%), naphthalene (9.94%), eucalyptol (8.11%), *α*- and *β*-pinene (3.31% and 1.8%, respectively), *β*-myrcene (1.49%), germacrene D (1.36%), and borneol (1.18%). In comparison, the major chemical constituents for MSEO were pulegone (37.16%), pyrazines (33.81%), limonene (11.19%), umbellulone (6.09%), camphor (4.27%), 3-carene (1.34%), 2-bornanone (1.2%), menthone (0.98%), 1-octen-3-ol (0.97%), *α*-pinene (0.88%), and thujone (0.60%) (Table 5).

## 4. Discussion

The present in vitro study showed the remarkable antioxidant, antidiabetic, and antibacterial properties of SOEO and MSEO, and it also showed the comparable antioxidant and antidiabetic superiorities of SOEO but the antibacterial superiority of MSEO. These observed favourable antibacterial, antidiabetic, and antioxidant potentials of the two tested EOs, as well as the variance in their levels, could largely be attributed to the synergistic effects of their bioactive principal constituents [34, 35].

MSEO and SOEO chemical compounds were reported by numerous studies [36–39]. These investigations showed the richness of SOEO by thujone, caryophyllene camphor, eucalyptol, *α*-pinene, *β*-myrcene, borneol, and *γ*-terpinene as main compounds and MSEO by pulegone, limonene, camphor, menthone, *α*-pinene, and thujone as main compounds.

For instance, pulegone, which herein is one of the major components detected in MSEO, has been recently described as a potential and novel inhibitor of bacterial growth and biofilm formation in multidrug resistant (MDR) bacteria by suppressing specific genes in susceptible bacteria [40]. Similarly, pyrazines are well-known as volatile organic compounds with broad-spectrum antimicrobial activity mediated by their diffusion into various bacterial structures and result in cell envelope disintegration and DNA destructing in susceptible bacteria [41]. Additionally, due to their high efficacy in combating MDR-bacterial pathogens at lower concentrations and minimal mammalian toxicity, pyrazines are prone to be applied in various aspects of food industry to prevent microbial spoilage and contaminations [41–44]. Some recent studies have also explored the unique bactericidal mechanism for each of 3-carene [45] and 1-octen-3-ol [46] by disintegrating cell membranes integrity and metabolic functionality of food- and nonfood-related virulent pathogenic Gram-positive and Gram-negative bacteria. At a constant line, naphthalene, which herein was among the major components of SOEO, is a well-known aromatic compound with potent antibacterial, antiviral, and antituberculous activities mediated by its covalent interactions with pathogen cellular proteins to produce potent cytotoxic and lytic effects on these pathogens [47, 48]. Most importantly, several naphthalene containing drugs with a broad spectrum of biological activities are approved by the FDA and are available as therapeutics [47]. Closely, eucalyptol has been latterly proposed as an excellent natural replacer for antibiotics due to its powerful bactericidal activity against a wide range of potential pathogenic Gram (+) and Gram (−) bacteria [49]. Moreover, eucalyptol has also shown remarkable synergistic interaction with traditional antibiotics such as amoxicillin and gentamicin, suggesting its combination therapeutic benefit in cases of MDR and mixed-bacterial infections [49]. Recently, *α*-pinene has also faced a specific attention in antimicrobial therapy. Besides its predominant bactericidal activities against antibiotic resistant *Staphylococci*, *Streptococci*, *Enterococci*, *Salmonella*, *Escherichia coli*, and *Pseudomonas* species, *α*-pinene has been identified as potential inhibitors for the bacterial efflux's pumps, which extrudes antibiotics out of bacterial cells and represents one of the main mechanisms contributing to MDR pathogenic bacteria [50–52]. Finally, the promising antimicrobial properties of thujone, caryophyllene, humulene, umbellulone, camphor, limonene, menthane, germacrene D, myrcene, and borneol, particularly against antibiotics-resistant microbial pathogens, have also been documented [53–55].

Diabetes mellitus (DM) is a highly complexed, chronic, metabolic disease caused by failure of pancreatic *β*-cells to produce sufficient insulin hormone alongside peripheral insulin resistance (IR) and characterized by persistent hyperglycemia and progressive metabolic and cellular changes [56, 57]. Nowadays, it has become more evident that progressive oxidative stress and mass production of reactive oxygen species (ROS) are among the ultimate contributors for pancreatic *β*-cell impairment and destruction, as well as in development of IR and multiple organ injury in diabetic patients [58]. Moreover, uncontrolled hyperglycemia, particularly postprandial hyperglycemia, is known as the most important risk factor for the generation of ROS and inflammation and act synergistically with oxidative stress to exacerbate *β*-cell failure, peripheral IR, and multimicro and macrovascular complications of DM. Hence, simultaneous controlling of hyperglycemia and oxidative stress is a paramount demand in DM therapy [59]. Despite the availability and the therapeutic benefits of several classes of antidiabetic-hypoglycemic agents, they are expensive, and their use is often associated with a wide range of side effects and nonproper control of patient's hyperglycemia. Therefore, the search for alternative antidiabetic therapeutic options, specifically for approaches based on medical plants products with potential antidiabetic and antioxidant properties, has gained specific importance to overcome the disadvantages of currently used synthetic antidiabetic compounds [60]. Herein, the antidiabetic potentialities of SOEO and MSEO were identified by their potent inhibitory effects on *α*-amylase and *α*-glucosidase enzymatic activities, in addition to their potent antioxidant capacity. Inhibition of *α*-glucosidase and *α*-amylase activities, which are the main enzymes responsible for gastrointestinal carbohydrates digestion and for starch hydrolysis into glucose preabsorption, is one of the most hopeful therapeutic targets in DM therapy to reduce postprandial hyperglycemia and prevent the absorption of dietary starch [60, 61]. Moreover, SOEO, which is rich in thujone, is known for its metformin-like antidiabetic effect, and oral administration of the leaves of *Mentha* plant was found to markedly suppress the blood indices of lipid peroxidation and increase the levels of enzymatic and nonenzymatic antioxidant variables, in patients with type II DM [62]. Such previous clinical observations of the antidiabetic usefulness of SOEO and MSEO, alongside our current findings, could also be primarily mediated by the augmenting interactions between their biological active components [63, 64]. In this regard, and as presented here, the chemical composition of the two EOs (Table 5) showed an enrichment in monoterpenoids compounds, such as pulegone, thujone, umbellulone, limonene, camphor, *α*- and *β*-pinene, menthone, borneol, and bornanone, which collectively have well-documented potent antihyperglycaemic, antioxidant, and anti-inflammatory properties [65, 66]. Additionally, monoterpenes have been reported to stimulate insulin release from pancreatic *β*-cells, reduce cellular oxidative stress, and modulate enzymes, proteins, and pathways that contribute to the development of IR and other pathological events, and thus, they were emerged as promising natural molecules to treat DM and a vast range of metabolic disorders [65]. Likewise, both humulene and caryophyllene have been documented as pluripotent free radical scavengers and *α*-glucosidase and *α*-amylase inhibitors [67, 68].

### 4.1. Study Limitations

In addition to the study's main findings, important limitations were inevitably identified, that should be addressed in the future. First, these findings are at the in vitro level, and to be more convinced, future in vivo studies in experimental rat and mice models of DM [59] and bacterial diseases [69] are essentially required, in which a second level of biochemical, molecular, and mechanistic analyses will be added. Second, the safety and toxicity profile and therapeutic index of these two EOs need to be determined in normal experimental animals to confirm the reliability of their clinical application in the future.

## 5. Conclusion

Findings of the present study revealed the chemical composition and antioxidant, antidiabetic, and antibacterial activities of SOEO and MSEO. The two tested essential oils inhibited *α*-amylase and *α*-glucosidase activities and showed potent in vitro antioxidant capacity. In addition, they exhibited significant bactericidal activity at lower concentrations against *Staphylococcus aureus*, *Staphylococcus epidermidis*, *Enterococcus faecalis*, *Listeria monocytogenes*, *Bacillus cereus*, *Escherichia coli*, *Salmonella typhimurium*, *Klebsiella pneumoniae*, and *Proteus mirabilis* bacterial strains, but weak activity against *Pseudomonas aeruginosa*. The observed favourable biological activities of these oils may primarily be attributed to the synergistic and/or additive effects of their bioactive chemical constituents. However, further studies are required to elucidate the present findings at the molecular and pharmaceutical levels and support their reliability for medical applications.

## Figures and Tables

**Table 1:** antioxidant activity of the essential oils (EOs) of *Salvia officinalis* and *Mentha suaveolens*.

Assay	Ascorbic acid^*∗*^	Trolox^*∗*^	*S. officinalis* EO	*M. suaveolens* EO
DPPH	10.73 ± 0.82	17.42 ± 1.85	93.67 ± 2.17^a,b,c^	53.19 ± 1.12^a,b^
ABTS	36.29 ± 1.74	35.64 ± 3.27	129.74 ± 2.11^a,b,c^	75.19 ± 1.80^a,b^
FRAP	18.95 ± 1.56	39.16 ± 2.14	112.41 ± 3.18^a,b,c^	69.48 ± 2.05^a,b^

DPPH, 2,2′-diphenyl-1-picrylhydrazyl radical scavenging assay; ABTS, 2,2-azino-bis-3-ethylbenzothiazoline-6-sulfonic acid radical scavenging assay; FRAP, Fe^+3^ reducing/antioxidant power assay. ^*∗*^Ascorbic acid and Trolox were used as antioxidant positive controls. Values are IC_50_ in *μ*g/mL and ^a,b,c^*p* < 0.05 vs. ascorbic acid, Trolox, and *M*. *suaveolens* EO, respectively.

**Table 2:** In vitro inhibitory effects of *Salvia officinalis* and *Mentha suaveolens* essential oils (EOs) on *α*-amylase and *α*-glucosidase enzymatic activities.

Assay	Acarbose	*S. officinalis* EO	*M. suaveolens* EO
*α*-Amylase (IC_50_ in *μ*g/mL)	396.42 ± 5.16	81.91 ± 0.03^a^	94.30 ± 0.06^a^
*α*-Glucosidase (IC_50_ in *μ*g/mL)	199.53 ± 1.12	113.17 ± 0.02^a^	141.16 ± 0.2^a^

^a^ *P* < 0.05 vs. the positive control (acarbose).

**Table 3:** In vitro antibacterial activities of the essential oils (EOs) of *Salvia officinalis* and *Mentha suaveolens* via the disc-diffusion method. Chloramphenicol and DMSO were used as a positive and negative control, respectively.

	*S*. *officinalis* EO (10 *μ*l/disc)	*M*. *suaveolens* EO (10 *μ*l/disc)	Chloramphenicol (30 *μ*g/disc)	10% DMSO (10 *μ*l/disc)
Gram-positive bacteria				
*Staphylococcus aureus*	28.0 ± 0.6	31.6 ± 0.6^a^	30.8 ± 0.6	—
*Staphylococcus epidermidis*	25.1 ± 0.5	27.5 ± 0.3^a^	28.7 ± 0.3	—
*Enterococcus faecalis*	20.2 ± 0.4	22.7 ± 0.4^a^	23.4 ± 0.3	—
*Listeria monocytogenes*	25.0 ± 0.3	29.6 ± 0.4^a^	27.5 ± 0.2	—
*Bacillus cereus*	26.8 ± 0.4	30.4 ± 0.3^a^	29.0 ± 0.2	—

Gram-negative bacteria				
*Escherichia coli*	18.6 ± 0.3	21.7 ± 0.2^a^	28.1 ± 0.3	—
*Salmonella typhimurium*	16.3 ± 0.2	18.7 ± 0.1^a,b^	21.0 ± 0.3	—
*Klebsiella pneumoniae*	17.6 ± 0.2	19.4 ± 0.2^a,b^	23.1 ± 0.2	—
*Proteus mirabilis*	17.1 ± 0.3	21.3 ± 0.1^a,b^	25.3 ± 0.5	—
*Pseudomonas aeruginosa*	8.1 ± 0.5^b^	9.2 ± 0.1^b^	—	—

Values are the mean zone of inhibition (mm) ± SD. (–), undetected activity. ^a,b^*P* < 0.05 vs. *S*. *officinalis* EO and chloramphenicol, respectively.

**Table 4:** Minimum inhibitory concentration (MIC), minimum bactericidal concentration (MBC), and MBC/MIC values of the essential oils (EOs) of *Salvia officinalis* and *Mentha suaveolens* against the ten tested bacterial strains.

Bacterial strain	*S*. *officinalis*			*M*. *suaveolens*			Chloramphenicol		
	MIC	MBC	MBC/MIC	MIC	MBC	MBC/MIC	MIC	MBC	MBC/MIC
*Escherichia coli*	3.12^a,b^	6.25^a,b^	2	1.56^b^	1.56^b^	1	4.0	4.0	1
*Salmonella typhimurium*	6.25^a,b^	12.5^a,b^	2	3.12^b^	6.25^b^	2	4.0	8.0	2
*Klebsiella pneumoniae*	3.12^a,b^	6.25^a,b^	2	1.56^b^	1.56^b^	1	4.0	4.0	1
*Proteus mirabilis*	3.12^a,b^	6.25^a,b^	2	1.56^b^	3.12^b^	2	4.0	8.0	2
*Pseudomonas aeruginosa*	25.0^b^	50.0^a,b^	2	25.0^b^	25.0^b^	1	64	64	1
*Staphylococcus aureus*	0.78^a,b^	1.56^a,b^	2	0.39^b^	0.39^b^	1	2.0	4.0	2
*Staphylococcus epidermidis*	1.56^a,b^	1.56^a,b^	1	0.78^b^	1.56^b^	2	4.0	4.0	1
*Enterococcus faecalis*	3.12^a,b^	3.12^a,b^	1	1.56^b^	1.56^b^	1	2.0	4.0	2
*Listeria monocytogenes*	0.78^a,b^	1.56^a,b^	2	0.78^b^	0.78^b^	1	2.0	2.0	1
*Bacillus cereus*	1.56^a,b^	1.56^a,b^	1	0.78^b^	1.56^b^	2	4.0	4.0	1

Values of MIC and MBC for the tested EOs are in mg/mL, while for chloramphenicol are in *μ*g/mL. ^a,b^*P* < 0.05 vs. *M*. *suaveolens* EO and chloramphenicol, respectively.

**Table 5:** Chemical composition of the essential oils (EOs) of *Salvia officinalis* and *Mentha suaveolens*.

No.	*Salvia officinalis* EO		*Mentha suaveolens* EO	
	Compound	%	Compound	%
1	Thujone	33.77	Pulegone	37.16
2	Caryophyllene	12.28	Pyrazines	33.81
3	Humulene	12.19	Limonene	11.19
4	Camphor	11.52	Umbellulone	6.09
5	Naphthalene	9.94	Camphor	4.27
6	Eucalyptol	8.11	3-Carene	1.34
7	*α*-Pinene	3.31	2-Bornanone	1.2
8	*β*-Pinene	1.8	Menthone	0.98
9	*β*-Myrcene	1.49	1-Octen-3-ol	0.97
10	Germacrene D	1.36	*α*-Pinene	0.88
11	Borneol	1.18	Thujone	0.60
12	Cyclopentane-3	0.96	o-Menth-8-ene	0.39
13	*trans*-*β*-Ocimene	0.60	*trans*-Calamenene	0.17
14	*γ*-Terpinene	0.51		

## Data Availability

The data used to support the findings of this study are included within the article.

## Abbreviations

ABTS: 2,2′-Azino-bis(3-ethylbenzothiazoline-6-sulphonic acid
ANOVA: One-way analysis of variance
*β*-cell: Beta cells in pancreatic islets
CFU: Colony forming unit
DM: Diabetes mellitus
DMSO: Dimethyl sulfoxide
DPPH: 2,2′-Diphenyl-1-picrylhydrazyl
FDA: U.S. Food and Drug Administration
FRAP: Ferric reducing/antioxidant power
GC/MS: Gas chromatography/mass spectrometry
IR: Insulin resistance
EO: Essential oil
IC_50_: The half-maximal inhibitory concentration
MDR: Multidrug resistant
MBC: Minimum bactericidal concentration
MIC: Minimum inhibitory concentration
MSEO: *Mentha suaveolens* essential oil
ROS: Reactive oxygen species
SOEO: *Salvia officinalis* essential oil
TTC: 2, 3, 5-Diphenyltetrazolium chloride
VS: Versus.
